# Revenue loss due to whale entanglement mitigation and fishery closures

**DOI:** 10.1038/s41598-022-24867-2

**Published:** 2022-12-13

**Authors:** Rachel Seary, Jarrod A. Santora, Desiree Tommasi, Andrew Thompson, Steven J. Bograd, Kate Richerson, Stephanie Brodie, Dan Holland

**Affiliations:** 1 Institute of Marine Sciences, University of California, Santa Cruz, CA USA; 2 Fisheries Ecology Division, Southwest Fisheries Science Center, National Marine Fisheries Service, National Oceanic and Atmospheric Administration, Santa Cruz, CA USA; 3 Environmental Research Division, Southwest Fisheries Science Center, National Marine Fisheries Service, National Oceanic and Atmospheric Administration, Monterey, CA USA; 4 Department of Applied Math, University of California, 1156 High Street, Santa Cruz, CA 95064 USA; 5 Fisheries Research Division, Southwest Fisheries Science Center, National Marine Fisheries Service, National Oceanic and Atmospheric Administration, San Diego, CA USA; 6 Fishery Resource Analysis and Monitoring Division, Northwest Fisheries Science Center, Newport Field Station, 2032 S.E. OSU Drive, Newport, OR 97365 USA; 7 Conservation Biology Division, Northwest Fisheries Science Center, 2725 Montlake Blvd E., Seattle, WA 98112 USA

**Keywords:** Socioeconomic scenarios, Conservation biology

## Abstract

Whale entanglements with fishing gear, exacerbated by changing environmental conditions, pose significant risk to whale populations. Management tools used to reduce entanglement risk, for example temporary area restrictions on fishing, can have negative economic consequences for fishing communities. Balancing whale protection with sustaining productive fisheries is therefore a challenge experienced worldwide. In the California Current Ecosystem, ecosystem indicators have been used to understand the environmental dynamics that lead to increased whale entanglement risk in a lucrative crab fishery. However, an assessment of socio-economic risk for this fishery, as in many other regions, is missing. We estimate retrospectively the losses from ex-vessel revenue experienced by commercial Dungeness crab fishers in California during two seasons subject to whale entanglement mitigation measures using a Linear-Cragg hurdle modeling approach which incorporated estimates of pre-season crab abundance. In the 2020 fishing season, our results suggest total revenues would have been $14.4 million higher in the Central Management Area of California in the absence of closures and other disturbances. In the 2019 fishing season, our results suggest ex-vessel revenues would have been $9.4 million higher in the Central Management Area and $0.3 million higher in the Northern Management Area. Our evaluation should motivate the development of strategies which maximize whale protection whilst promoting productive, sustainable and economically-viable fisheries.

## Introduction

Conflicts arising from fishery bycatch (incidental capture of non-target species such as marine mammals, seabirds and turtles) threaten recovery and conservation of protected species and bring about socioeconomic impacts for coastal fishing communities^1–3^. Whilst there is evidence of some whale populations rebounding in recent years^4–7^, the problem of entanglements in fishing gear still exists and poses significant risk to many endangered and threatened whale populations globally^8–14^. Environmental impacts from climate variability and change have exacerbated whale entanglements in recent years in particular fisheries^15–19^. These entanglements often involve species that are legally protected in the United States under the Federal Endangered Species Act or Marine Mammal Protection Act and thus result in temporary fishery closures. In such fisheries, building partnerships between fishery managers and fishing communities is important for reducing both whale entanglement risk and economic impacts of whale protection. Developing mitigation strategies to reduce whale entanglement risk is now a priority of many fixed gear and trap-based fisheries that utilize vertical lines as these gears are most commonly involved in bycatch of whales. Strategies involve using indicators of risk such as metrics of environmental conditions, marine mammal densities and fishery specific metrics, such as monitoring target species and fishing behavior^20,21^. Faced with these challenges, fishery managers often apply mitigation measures that either reduce the number of traps and vertical lines, or set guidelines on opening and closing a fishery to minimize spatiotemporal overlap between peak periods of protected species occurrence in fishing grounds and fishing activities^13,14,22–26^. However, development and enactment of risk mitigation for protected species often lack a risk assessment to assess the economic viability of the fishery facing enhanced mitigation^27^.

The California Dungeness crab (*Metacarcinus magister* or *Cancer magister*) pot fishery is one of the most lucrative and important fisheries on the US West Coast^28^, bringing in more than $80 million in ex-vessel revenues in recent years^29^. The California Dungeness crab fishery typically operates from mid-November through mid-July^30^, with highest potential conflict with whales in either the season opener or during spring and summer, when whales migrate to and from seasonal foraging and overwintering grounds. Since 2014, the California Dungeness crab fishery recorded a marked increase in the number of reported whale entanglements which was attributed primarily to a prolonged marine heatwave and delayed season opener due to contamination of crab from a Harmful Algal Bloom (HAB)^17^. Humpback whales (*Megaptera novaeangliae*) make up 58% of reported entanglements in California between 2000 and 2021 but entanglements in the region have also involved gray (*Eschrichtius robustus*) and blue whales (*Balaenoptera musculus*)^31^.

**Figure 1:**
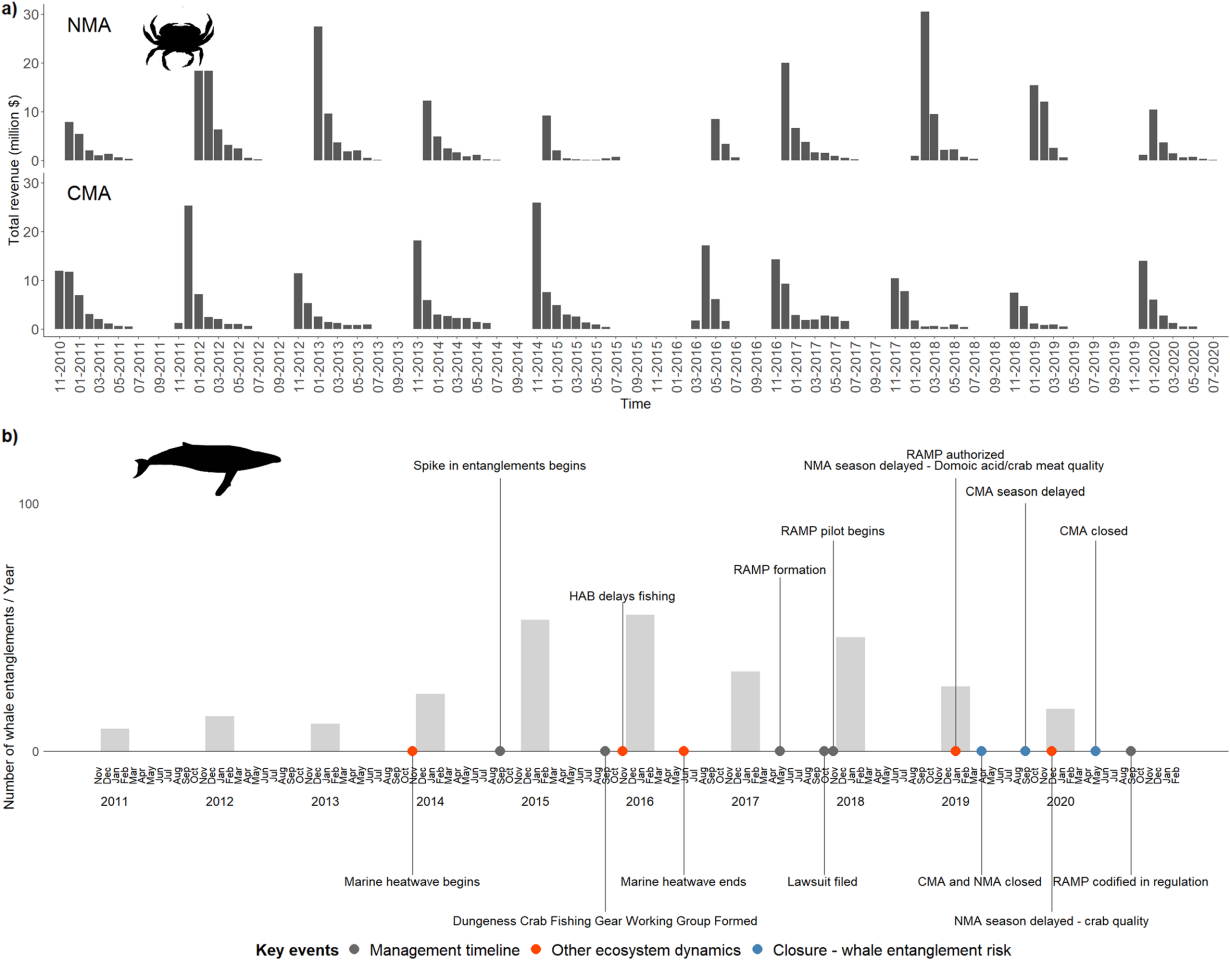
Timeline showing (**a**) total monthly ex-vessel revenues from Commercial Dungeness crab fishing in the Northern and Central Management areas of California and (**b**) the number of whale entanglements recorded in California (bars represent the number of reported entanglements of all whale species in each year) and the timeline of biological and management events influencing the fishery. Data points representing less than 3 participants have been removed for confidentiality. This figure was created in R Studio (Version 4.0.3)^64^. Images were obtained from PhyloPic (http://phylopic.org/), created by Chris Huh (Humpback whale) and Harold N Eyster (Dungeness crab), and are both available for reuse under the Creative Commons Attribution 3.0 Unported license (https://creativecommons.org/licenses/by/3.0/).

The crab fishery has worked to reduce the number of whale entanglements by developing and applying a Risk Assessment and Mitigation Program (RAMP). The RAMP, informed by the Dungeness crab Fishery Gear Working Group, is composed of crab fishers, resource managers, environmental non-governmental organization members and scientific advisors^32^ and is now a legal mandate for managing the fishery^33^ (Fig. 1). Starting in the 2018–2019 fishing season, risk mitigation measures are used to reduce whale entanglements, and have been implemented through either a delayed opening and/or early closure. The RAMP largely focuses on mitigating entanglement risk by monitoring environmental conditions and whale concentrations within management areas. It currently does not include an economic impact risk component, nor were socio-economics considered during its development. Humpback whales feeding or travelling along the California coast include individuals from distinct population segments that are federally listed endangered or threatened species and are required to be protected under the Federal Endangered Species Act of 1973. However, under the RAMP, socio-economic impact can be considered (should information be available) if two regulatory actions would equally reduce entanglement risk^33^. In addition to area closures, the fishery has acted to reduce whale entanglements through an abandoned trap gear retrieval program and permanent mitigation measures such as a best practice guide which sets limits to trailer buoy numbers and the length of surface lines^34^.

Under new RAMP regulations^33^, whale entanglement risk is assessed using data on marine life concentrations before the California Dungeness crab fishery opening and continuously until the season ends. Together with increasing whale populations and changing ocean conditions, increased risk of entanglement and more stringent regulations suggest that fishery closures and delays may become more frequent in coming years^17^. Prior to whale entanglement mitigation closures, a multi-month delayed opening caused by a marine heatwave-induced HAB in the 2016 season caused significant economic impacts to fishers and the wider community, requiring $25 million in federal disaster financial assistance^35–38^. New regulations imposing closures due to whale entanglement risk could bring similarly negative consequences for coastal fishing communities, but these potential losses have not been measured.

The Dungeness crab fishery has long been considered a reliable and economically lucrative fishery for US West Coast fishing communities^39–41^. Populations of Dungeness crab fluctuate markedly from year to year and are driven by density-dependent biological mechanisms as well as exogenous environmental perturbations^42^. Sea surface temperature, sea surface height and upwelling are among ecosystem level physical forces driving Dungeness crab populations, which are common drivers amongst other species in the CCS such as salmon populations^43^. Thus, the status of Dungeness crab fishery is linked to the status of other CCS commercial fisheries through their relative responses to common physical drivers. The fishery is also strongly embedded within US West Coast fishery networks, with a high proportion of all fishers gaining revenue from Dungeness crab fishing while also participating in a number of other fisheries and therefore understanding impacts to this fishery is relevant to the management of other fisheries through cross-participation in the CCS^28,44^. Estimating losses from the Dungeness crab fishery resulting from past closures due to whale entanglement risk will benefit ongoing risk mitigation planning, and will help to inform potential impacts of future events.

Our objective is to estimate retrospectively any revenue losses that occurred within the past 2 California Dungeness crab fishing seasons as a result of fishery closures prompted by increased whale entanglement risk. Here, we estimate lost revenues by applying an economic impacts model^38^ designed to measure revenue loss at the individual vessel level, which we then aggregate to the fishery level for two fishing management areas of California. We use hurdle models of crab revenue that estimate both the probability of a vessel participating in the fishery and the expected ex-vessel revenue contingent on participation. Models were fit with historical landings data, from 2011 to 2018, which included estimates of pre-season Dungeness crab abundance and vessel characteristics including frequency of participation, fishing strategy concentration index (as measured by the Herfindahl–Hirschman Index (HHI)), vessel size, latitude of fishing, proportion of revenues from Dungeness Crab and a binary variable which indicated whether a vessel switched between management areas within a fishing season. Retrospective loss estimates are determined by comparing observed to predicted revenues at the vessel level for the 2018–2019 and 2019–2020 fishing seasons. With this approach, which assesses the cumulative revenues through the season, we seek to quantify whether delays or closures implemented for whale entanglement regulation affect an overall seasons’ revenues. In measuring revenues cumulatively throughout the season, associated losses include revenues lost due to other disturbances to the season, such as crab quality delays, harmful algal blooms and whale entanglement risk. Quantifying the cost of closures will allow socio-economic impacts to be considered alongside ecological concerns within the decision-making process.

## Results

We quantified direct economic impacts arising from delayed opening and early closures prompted by whale entanglement risk mitigation as well as crab meat quality concerns for two fishing seasons (2018–2019 and 2019–2020, subsequently referred to as 2019 and 2020 seasons) in the California Dungeness crab fishery (Fig. 1). Although whale entanglements have declined on the US West Coast since peaking during the large marine heatwave in 2014–2016 (Fig. 1b), several delayed opening and early closures of the fishery were enacted to minimize whale entanglement risk (Fig. 1b). This provided a unique opportunity to assess economic impacts in a rapidly changing fishery and management landscape.

**Figure 2:**
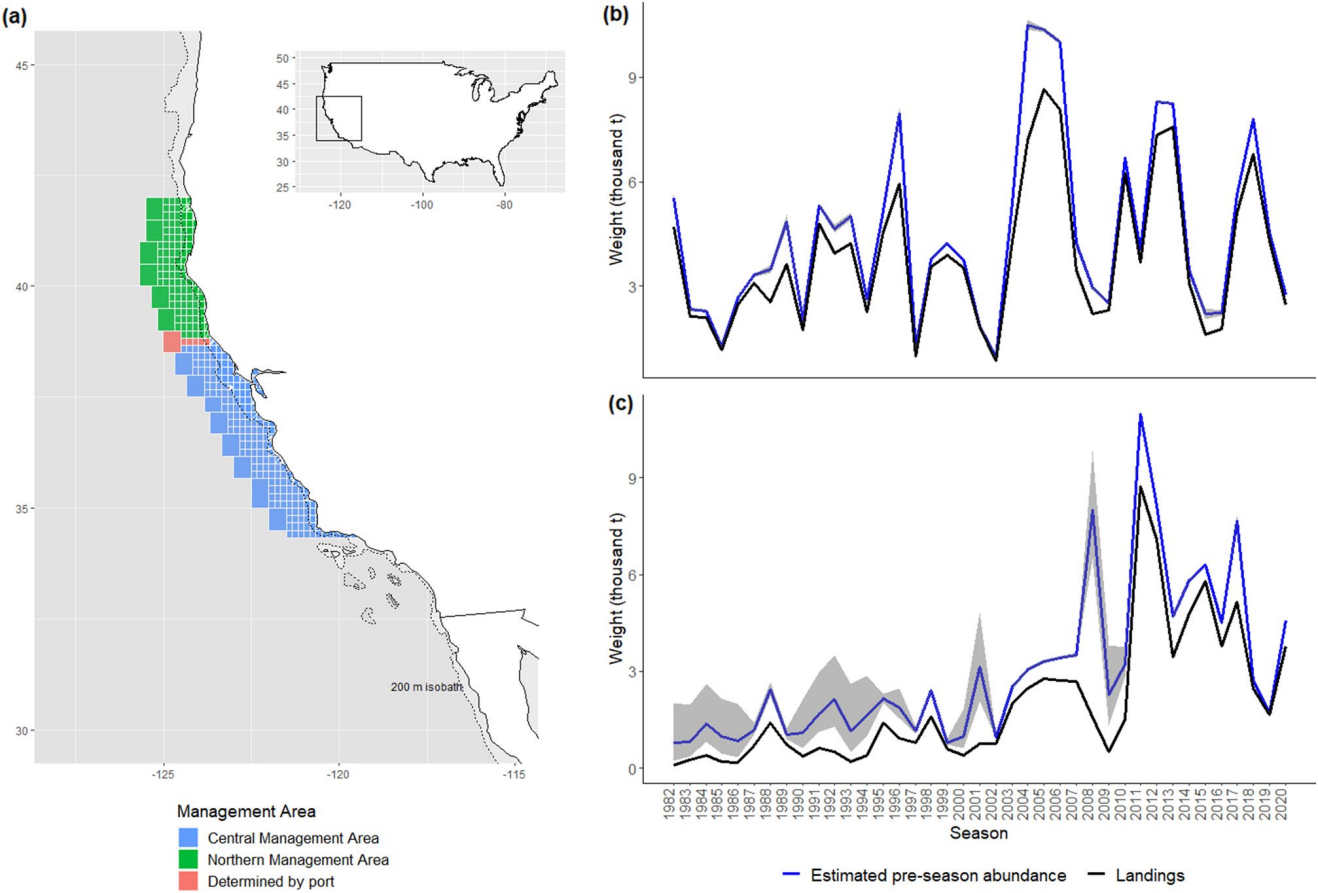
(**a**) Location of the California Commercial Dungeness crab fishery management areas in the California Current Ecosystem and its location with the USA and estimates of pre-season abundance of legal sized male Dungeness crab by season for (**b**) Northern and (**c**) Central California using a hierarchical depletion estimator model from Richerson et al. (2020)^62^. This figure, including the map in 2a, was created in R Studio (Version 4.0.3)^64^. The basemap and 200 m isobath were obtained from the rnaturalearth package (Made with Natural Earth. Free vector and raster map data @ naturalearthdata.com).

Our models incorporated pre-season crab abundance, which was estimated for the two management areas (north management area, NMA; and central management area, CMA; Fig. 2). Pre-season abundance of legal sized male Dungeness crab was estimated at 4.54 and 1.72 thousand tons for the 2019 season and 2.75 and 4.55 thousand tons for the 2020 season for the NMA and CMA respectively (Fig. 2b,c). All covariates except for the mean percent of crab within a vessel’s total revenues during the baseline period (2011–2018 seasons) had a significant effect (*p* < 0.05) on the selection (participation) model in the NMA. In the CMA, all covariates other than mean latitude of catches had a significant effect on participation (Supplementary Table S1). Fishing strategy concentration (HHI) as well as mean latitude of catches and vessel length had a negative relationship with participation in both regions, suggesting higher probability of participation by vessels with diverse strategies, fishing at lower latitudes within regions and with smaller vessels. (Supplementary Table S1). Mean latitude of crab catches however was not a significant variable in participation in the CMA.

In the revenue model, all covariates other than the mean percent of crab within total vessel revenues were significant for the NMA (Supplementary Table S1). All covariates other than fishing strategy concentration and switching had a positive sign with crab revenues. Participating in fishing in both management areas within a given season had a negative impact on total crab revenues in the NMA. This switching behavior had a negative relationship but statistically non-significant relationship with crab revenues in the CMA. All other covariates were significant in the crab revenues model for the CMA. Of the significant covariates, mean latitude and the diversity index had a negative relationship with crab revenues while all others showed positive relationships with crab revenues. Deviance explained by crab models, calculated using an analogue for R^2^, was 0.39 for the NMA model and 0.28 for the CMA model. The model performed well overall at predicting revenues at the fishery level but over-estimated revenues for vessels that did not participate in the two seasons studied and underestimated revenues for some high earning vessels (see Supplementary Fig. S1a–d). The hurdle model includes revenue estimates of all vessels in the fishery, including those that did not fish in the seasons for which we predict revenues, to provide an accurate total revenue estimate for the entire fishery. This accounts for vessels that chose not to fish due to the closures. These values however are less useful in describing the mean losses at the vessel level across vessels that actively participated as the calculated mean is skewed by the extremes of the distribution. Therefore, when presenting revenue losses at the vessel level, we report the 25th–75th percentile of predicted revenues to remove the disproportionate influence of the extremes of the skewed distribution on the average expected losses.

**Figure 3:**
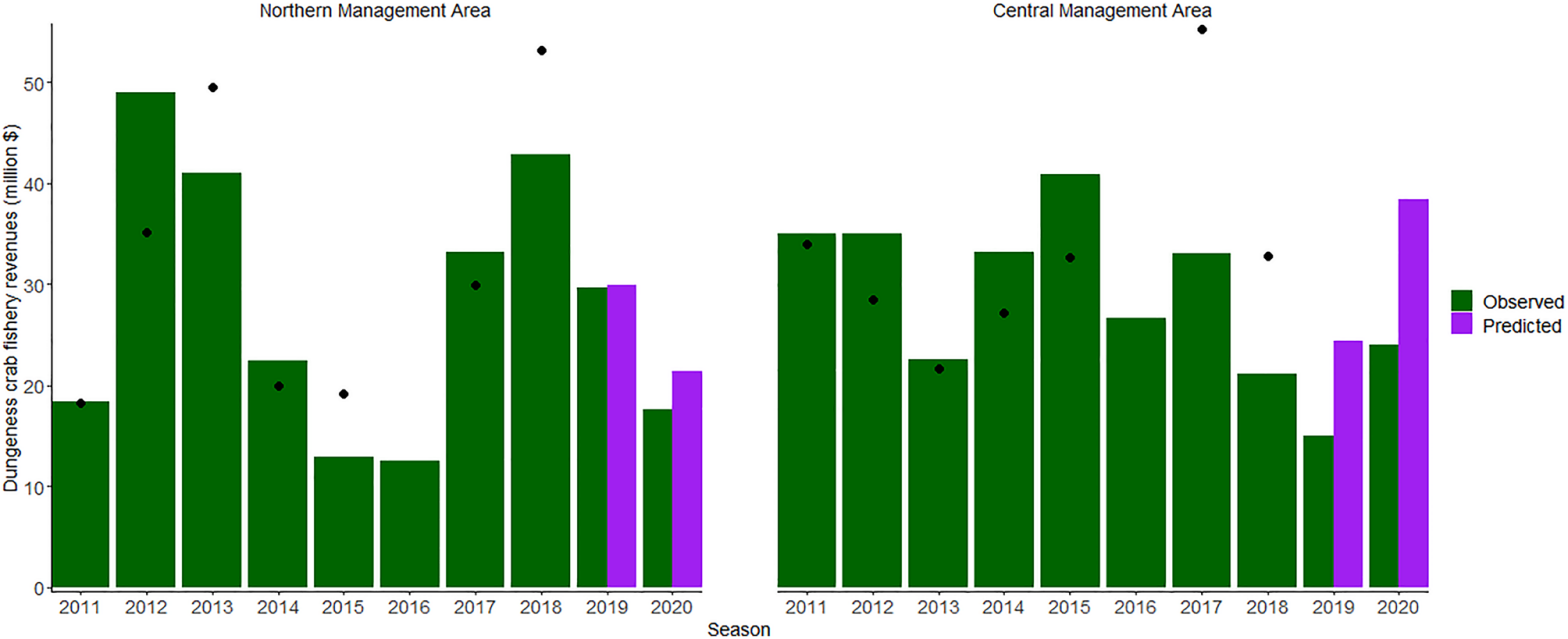
Observed and retrospectively predicted revenues at the fishery level for the Commercial Dungeness crab fishery in California, within the Northern and Central Management Areas. This figure was created in R Studio (Version 4.0.3)^64^. Points show out-of-sample predictions of Dungeness crab revenue and demonstrate possible model prediction error of predictions made for the 2019 and 2020 fishing seasons.

In the CMA, the model predicts that the total crab fishery revenue would have been $9.4 million higher in the 2019 season and $14.4 million higher in the 2020 season in the absence of closures and other disturbances (Fig. 3, Table 1). In the NMA, model results suggest that fishery revenues would have been $0.3 million higher than observed revenues in 2019 and $3.9 million higher in 2020 (Fig. 3, Table 1). In the 2020 season in the NMA no whale entanglement mitigation was enacted so losses represent other factors influencing revenues such as the impact of COVID-19 on markets (NMFS 2021)^39^.

**Table 1:** Observed, predicted and loss estimate values (million $) for revenues from the Commercial Dungeness crab fishery in California.

	2019 season			2020 season		
	Observed total fishery revenue	Predicted total fishery revenue	Predicted loss in total fishery revenue	Observed total fishery revenue	Predicted total fishery revenue	Predicted loss in total fishery revenue
Northern management area (n = 400)	29.56	29.84	0.28	17.55	21.42	3.87
Central management area (n = 525)	15.01	24.38	9.37	24.00	38.43	14.43

N represents the number of vessels included in the analysis.

At the vessel level in the CMA, based on the 25th to 75th percentile of predicted revenues, the model predicted that vessel level revenues would have been on average $7.8 (SD $39.57) thousand higher in the 2019 season and on average $17.70 (SD $58.04) thousand higher in the 2020 season. At the vessel level in the NMA, based on the 25th to 75th percentile, the model predicted that vessel level revenues would have been on average $1.22 (SD $62.00) thousand higher in the 2019 season and $9.73 (SD $46) thousand higher in the 2020 season.

**Figure 4:**
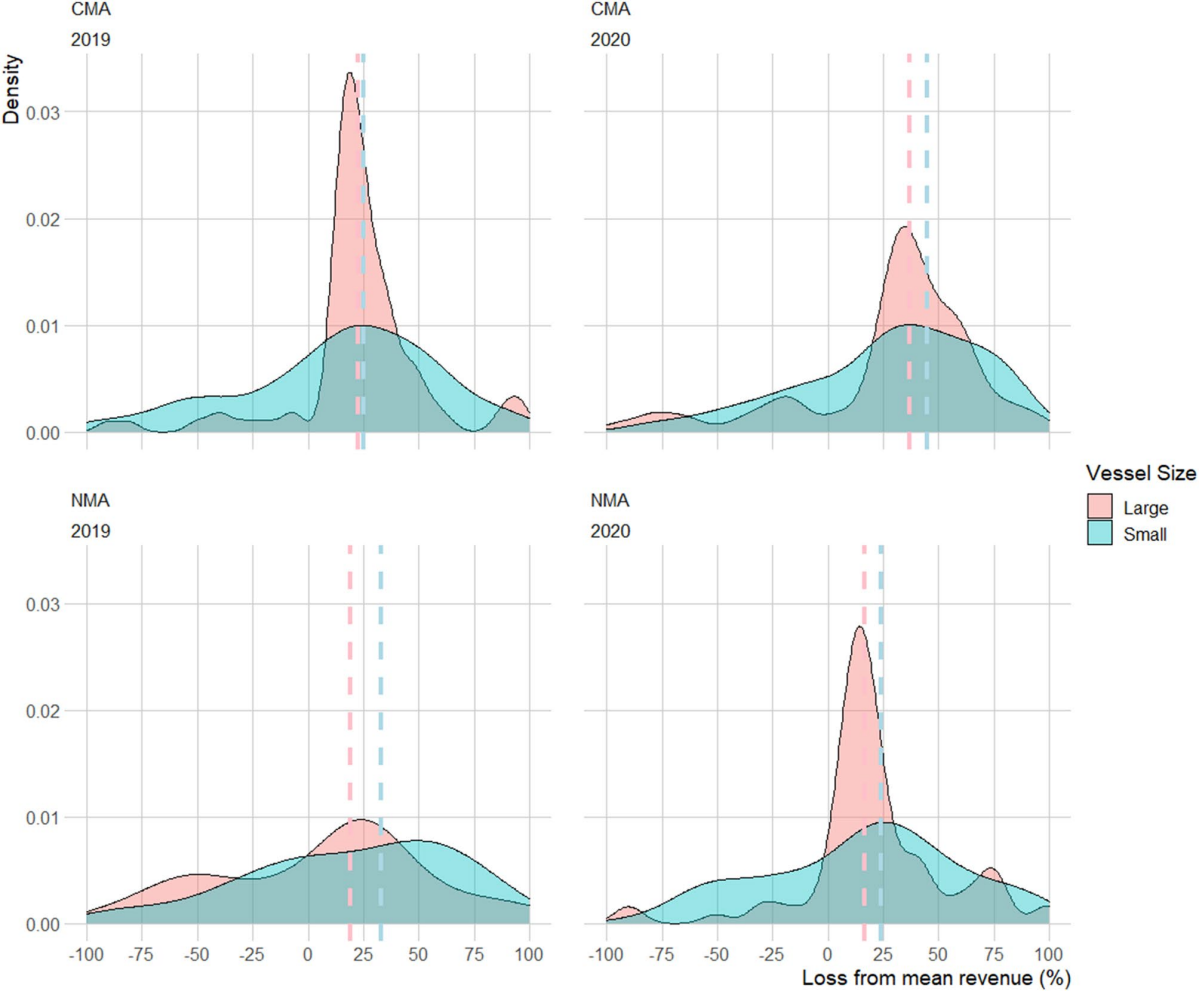
Density plots of estimated percentage ex-vessel revenue loss as a proportion of a vessels mean annual revenue (2011–2018) by small and large vessels in the Northern and Central Management Areas for the 2019 and 2020 fishing seasons. Median revenue losses are shown with dashed lines. Predicted revenues from within the 25th to 75th percentile are included. Negative numbers on the X axis indicate no revenue loss. The X axis has been restricted to between – 100 and 100 for plotting purposes.

As large and small vessels may be impacted differently by fishery interventions^45^, and there is large variability in vessel level revenues across the fishery, we also investigated losses predicted for small (< 40 ft) and large vessels (≥ 40 ft, the commonly used length cutoff for vessels in this fishery^45,46^) separately. Median estimated revenue losses were similar between small ($22 thousand in 2019, $14 thousand in 2020) and large ($21 thousand in 2019, $17 thousand in 2020) vessels in the NMA. However, estimated losses as a proportion of a vessels mean historical revenues were higher for small (median of 33% in 2019, and 24% in 2020) than large vessels (median of 19% in 2019, 16% in 2020) (Fig. 4).

In the CMA, revenue losses were larger for large vessels (median of $27 thousand in 2019 and $39 thousand in 2020) than small vessels (median $18 in 2019 thousand and $33 thousand in 2020). However, as a proportion of a vessels mean historical revenues, small vessels were estimated to have incurred larger percentage losses (median of 25% and 45% in 2019 and 2020) than large vessels (median of 23% and 37%) (Fig. 4).

Predicted fishery revenues in historical baseline (non-closure) years (in-sample predictions) were on average $0.47 (SD 5.79) million higher for the NMA model and $0.61 (SD 8.35) million higher for the CMA model than observed revenues suggesting some model error or unaccounted for variability. With the exception of 2019 predictions in the NMA, our predictions of losses during closure years are larger than the average model error. This possible error should be considered in the case of small revenue losses (e.g. NMA 2019) and as a proportion of larger predicted revenue losses. Average prediction residuals (observed-predicted revenue) at the vessel level are shown in (Supplementary Fig. S1c). We also provide out-of-sample prediction results. These were computed by iteratively holding out one year of data, fitting the model to the remaining data, and then making predictions for the held-out year (Supplementary Table S2). Out-of-sample predictions typically over-predicted revenue in the NMA and under-predicted revenue in the CMA, with overpredictions larger in magnitude (Supplementary Table S2). In the CMA, out-of-sample prediction errors ranged from a $22 million loss to a $8 million gain. Out-of-sample prediction errors in the NMA were smaller, ranging from a $10 million loss to a $14 gain. The largest out-of-sample prediction error was during 2017 in the CMA. This exceeded our prediction of a closure loss for both the 2019 and 2020 seasons, indicating that our predictions are quite uncertain. As the 2019 and 2020 seasons were predicted “out-of-sample”, some overestimation of revenues, and thus of losses may have occurred (Supplementary Table S2). However, our predictions in 2019 and 2020 do align well with revenue predictions based on a 5-year average (Supplementary Table S3), as is commonly done to calculate disaster assistance, which builds additional trust in our prediction models.

## Discussion

Whale entanglements in fishing gear threaten whale populations, seafood production and long-term sustainability of commercial fisheries. While multiple mitigation strategies to reduce entanglements exist, there has been minimal consideration of the economic impact of these strategies. Here, we estimated retrospective losses to ex-vessel revenues for one of California’s most lucrative fisheries. Overall, we found fishery closures decreased ex-vessel revenue, with results showing some uncertainty due to large model prediction error. Regional differences in losses revealed interesting trends in the capacity for the fishery to recoup costs. For example, in the NMA, relatively small losses at the fishery level were predicted ($0.3 million in total) for the 2019 season despite an early closure to the season due to whale entanglement risk.

NMA fishers collectively were able to meet predicted revenue for the season despite a shortening of the fishing 2019 season. In the 2020 season however, the NMA did not experience disturbances due to whale entanglements but larger ex-vessel losses (of $3.9 million) were predicted. This suggests that other disturbances such as a delay to the season due to crab meat quality, lost fishing opportunity related to the COVID-19 pandemic, or other unknown factors, had an influence on ex-vessel revenue during the 2020 season. While most of the 2020 season landings in the NMA occurred before COVID-19 arrived in the US, there is evidence that prices in latter part of the season may have been depressed due to loss of export markets for live crab^47^.

In the CMA however, despite landing the majority of crab available during the 2019 season (see Fig. 2c), losses of $9.4 million were experienced across the fishery. While total fishery catch was not greatly reduced, closure to the fishery in the spring may be responsible for revenue losses through other mechanisms (e.g. price). In the 2020 season, whale entanglement risk substantially shortened the fishing season in the CMA, through a delay at the beginning of the season and an early closure in the spring. Estimated losses were largest ($14.4 million) during this season. It is likely that the COVID-19 pandemic was also responsible for some of this estimated loss in the CMA in the 2020 season^47^. Our model did not control for impacts of the COVID-19 pandemic. However, price trends suggest that that price of Dungeness Crab in California was not affected until mid-March 2020, at which point the fishery had caught 92% of the seasons catch (see Supplementary File S2). Prices then returned to normal levels in mid-May. If we apply extrapolated prices between mid-March and mid-May by replacing observed prices with linearly increasing prices by week, revenues would have been $753,754 higher in total across the fishery. This rough estimate suggests we can attribute 4.1% of overall estimated revenue losses during the 2020 season to COVID-19 impacts, with the caveat that we do not know what prices would have been in the absence of the pandemic. A counterfactual approach has been used to disentangle multiple stressors to infer causal impacts of management interventions elsewhere^48^, however as these closures, and the COVID pandemic, potentially impacted all fishers in the California Dungeness crab fishery, there are no control groups available for comparison and therefore this approach would not be appropriate.

Closures and other disturbances appear to have been less impactful in the NMA and high price for Dungeness crab may have contributed to the ability of vessels operating in the NMA to withstand disturbances (Supplementary Fig. S2). Prices were particularly high during the summer portion of the season in 2020 during which time the CMA was closed to Dungeness crab fishing (Supplementary Fig. S2). The NMA did not experience closures due to whale entanglement during 2020 and was predicted to have lower than average pre-season abundance (lower catch potential) during 2020 (see Fig. 2.b), while the CMA was predicted to have high catch potential for 2020 (Fig. 2.c), therefore differences in management measures implemented, and seasons’ catch potential, also contributed to differences in losses estimated.

The CMA also experienced high prices, including decadal high prices for crab during the November–December of the 2019 fishing season (Supplementary Fig. S2). However, losses observed overall across the two seasons suggest the fishery, unlike the NMA, did not get much overall benefit from the high price in 2019 or the high pre-season abundance of crab (i.e. catch potential) estimated for the 2020 season in the CMA. A number of factors may have contributed to a poor season in the CMA including catchability or biology of Dungeness crab as well as external factors such as the COVID-19 pandemic behavioral choice factors, for example deciding not to fish^45^. Temporally shifting or reducing the opportunity for participation through closed periods due to whale entanglement risk may have exacerbated other impacts on revenues in the CMA which were not as impactful on revenues in the NMA.

The high variability in estimated economic impacts per vessel reported here demonstrates that closures did not affect all vessels equally, similarly to impacts observed following a climate related harmful algal bloom in the 2016 season which were variable by vessel size and between communities^45^. The estimated losses we present at the fishery level in the NMA and CMA may therefore be underestimated, or overestimated, for particular groups of vessels within those management areas. This reflects the diverse nature of the Dungeness Crab fishery in behaviour and fishing strategy and highlights the importance of capturing impacts at finer scales than the fishery level alone.

### Limitations to the estimation of closure impacts

A limitation of the hurdle model is that there are other latent factors influencing fishery participation and revenues that our model does not incorporate, particularly those determining fisher behavior such as fuel price, shipyard backlogs and market demand. A behavioral choice model, for example one that incorporates location or fishing alternative choice given a closure^50–52^ would be a potential method to better understand how spatial management strategies affect fisher behavior and is recommended as a future analysis to assess trade-offs involving socio-economic risk. Our results, reporting losses from Dungeness crab fishing revenue only, also do not account for the ability of some fishers to mitigate revenue losses by participating in other fisheries. Dungeness crab fishing is highly connected within west coast fishery participation networks^44,45^. Thus, it is important to note that our results for the 2019 and 2020 seasons present only losses from Dungeness crab fishing and may overestimate total annual revenue losses by some vessels that are able to mitigate impacts with participation in other fisheries.

The model, predicting out-of-sample, over-estimated revenues in recent years suggesting that our predictions of revenues may also be over-predicted. An improved estimation at the vessel level, given some over-estimation of vessels that did not fish, could be investigated through a selection model approach rather than a two-part model approach^54^. However, two-part models are most appropriate for estimation of conditional (actual) outcomes as was intended here rather than unconditional (potential) outcomes and they do not require separate drivers for the selection and estimation model, which we did not have available^54^. When the impacts of policy interventions are difficult to disentangle from other impacts, approaches such as a counterfactual synthetic control^48^ approach could be used to separate the impacts of the policy alone. In this context, however, it is useful to report the cumulative impact of disturbances given that these disturbances (e.g., delays due to crab quality, harmful algal blooms) happen frequently and therefore the closures will rarely happen in isolation.

Whilst there are limitations to our approach, revenue predictions presented here offer more insight compared to predicting revenues based only on a 5-year average of total fishery revenues (Supplementary Table S3) as is commonly conducted to calculate disaster assistance requirement, as our analysis includes an estimation of crab abundance as well as historical vessel level data in its estimation. Accounting for the influence of crab abundance is critical in this fishery given abundance is highly variable and the majority of fishable biomass is taken each year. Estimation of revenue at the individual vessel level allows for consideration of fishery heterogeneity (e.g., by vessel size). Revenues calculated on a 5-year average would suggest total California Commercial Dungeness crab fishery revenues would have been $10.62 million higher than observed in 2019 and $12.73 million higher than observed in 2020 (Supplementary Table S3). Thus, revenues estimated on the 5-year average suggest that losses would have been $0.97 million higher than our model prediction across the fishery for 2019 and $5.56 million lower than our model prediction for 2020. Our predictions suggest that delays and closures due to whale entanglement mitigation and other disturbances in to the 2019 and 2020 seasons were similar to the impact of closures due to the HAB in the 2016 season, which were estimated at $13.6 million in losses from Dungeness Crab revenues across the fishery^38^.

### Economic cost of mitigation

Many strategies that prevent fishery interactions with marine mammals exist, including gear reductions or modifications, depth limitations and dynamic or seasonal time-area closures^13,14,22–26,55^. Whilst the fishery does implement pro-active gear modification measures set out in the best practices guide^34^, only two management intervention options were enacted in the 2019 and 2020 seasons to mitigate against entanglements of marine life with Dungeness crab gear; delays to the start of the crab season in the winter and early closures in spring due to overlap with whale distribution in fishing grounds. These delays and closures can have differential impacts on the fishery as the fishing season is not heterogeneously prosperous. An example is that closures during the holiday season (Nov–Dec) when Dungeness crab is traditionally consumed can cause substantial lost revenue opportunity for fishers at a time when price and demand are highest^35,49^. The fishery operates as a derby in which the majority of revenues are made in the first month of the fishery being open. The strong seasonal dynamics of the Dungeness crab fishery, largely driven by rapid depletion of legal sized crab, mean that the timing of management actions can have important impacts on fishing revenues. Across the fishery, based on observed vessel level revenues during the 2011–2018 baseline period, vessels earned an average of 62.33% (SD 24.04) of annual ex-vessel revenue during the first month of the season (15th Nov–15th Dec for the CMA/1st Dec–31st Dec for the NMA). After April 1st, vessels on average earn 10.54% (SD 18.98) of annual ex-vessel revenue. This average, based only on vessels that historically have actively participate past April 1^st^, (283 vessels in the NMA, 346 vessels in the CMA) rises to 20.36% (SD 13.37) of ex-vessel revenue. Thus, while the majority of the overall fisheries revenue is taken at the start of the season, an April 1^st^ closure could still have a substantial impact on the revenues of active fishing vessels in the spring. Determination of economic risk for the fishery, at a minimum, should consider timing of closures in addition to total revenue losses, in order to quantify losses that will be felt at the individual vessel level. We suggest further research to investigate how closures affect different groups of fishers through stakeholder participation.

Socio-economic impacts from whale mitigation measures could permeate into communities further than our analysis (based on ex-vessel revenue only) conveys^35–37,49^, and further investigation into these community level impacts is necessary to understand and sustain an equitable fishery supply chain even where there is no absolute revenue loss. Some of the communities influenced by whale entanglement mitigation in California rely heavily on ocean resources for employment, through fishing occupations but also through hospitality and tourism. Managing this issue in a way that minimizes the burden on resource dependent communities is strongly in line with the objectives set out in the UN Sustainable Development Goals (SDG’s), especially SDG 14 (life below water) but also related goals such as human well-being, reducing inequality and reducing the impacts of climate change^56^.

### Management Implications

Balancing socio-economic impacts against whale entanglement risk is challenging given the legally protected status of whale populations. However, potential economic losses reported here should motivate the development of mitigation measures (through cooperative innovation between industry, researchers and managers) that allow fishery production to be optimized whilst ensuring successful whale protection. At present, entire management areas, which constitute large regions of the coast, are closed in response to whale entanglement risk in California. Investigating how to minimize the spatiotemporal footprint of closures, such as by defining high risk zones dynamically based on fine-scale information of whale density and fishing effort, could provide an alternative mitigation structure. This could better consider the economic and conservation trade-offs while still being sensitive to changing environmental conditions. The introduction of dynamic zone closures, often broadly referred to as dynamic ocean management, has been demonstrated to reduce risk whilst minimizing lost fishing opportunities^12,26,57,58^, especially when environmental variability is high or species have a dynamic distribution^59^. Moreover, analysis of policy instruments to reduce whale entanglements with the American lobster fishery on the US Northeast coast found that economic costs of risk reduction could be 20% lower when mitigation decisions considered fishing opportunity costs alongside non-monetary benefits (biological risk), compared to non-monetary benefits alone^12^. This is promising for the implementation of such strategies in the California Current System.

The caveat of this strategy is that dynamic zone closures require spatially and temporally explicit information on whale density and fishing effort which can be costly to attain. The use of ropeless gear has also been suggested as an alternative whale entanglement mitigation measure that requires further research and development before being initiated as an alternative regulatory tool^60^. The costs of monitoring or technical advancements however may outweigh the financial and societal cost of fishery closures. Revenue losses for Dungeness crab estimated here for the 2019 and 2020 seasons are on par with losses experienced during the HAB period. During the delays to the 2016 fishing season an estimated $26.1 million was lost from ex-vessel revenues from all species that crab fishers target, including $13.6 million from Dungeness crab alone^38^, requiring $25 million in government aid. Whale mitigation under the RAMP regulation will potentially delay or close the fishery year after year with uncertain economic impact that cannot be sustainably resolved with government aid. Development of tools to mitigate against economic loss while achieving whale protection will be necessary to come to a sustainable solution. This can only be achieved by first including economic loss in risk assessments. Doing so may also provide balance to partnerships between fishery managers and fishers.

Regulators are obligated to protect Humpback whales, blue whales and Leatherback turtles using the best available science^33^. In this fishery, current triggers to open and close are based on a range of factors, but thus ultimately depend on the number of whales present within a management region^33^. Regulators have a number of alternative regulatory options available to them, which include depth restrictions, gear restrictions or modifications and fleet advisories, if they can offer the same level of whale protection^33^. Yet, the RAMP process lacks the socio-economic information needed to consider the socio-economic risk of regulatory actions, and that of the alternatives, to the fishing community. Results presented here highlight that the economic effects and that risk to fishing communities should be considered when designing whale entanglement mitigation programs^33^. Having this economic information will facilitate the ability of managers, as set out in the RAMP regulation (subsection d4)^33^, to consider the socio-economic impact if deciding between management measures that equivalently reduce entanglement risk.

We have used two fishing seasons as an example of the economic impacts of these new whale entanglement regulations which will be implemented each year going forward. Synthesis of ex-vessel revenues is not a complete picture of the socio-economic impacts of regulations, but it provides a starting point for protecting both whales and fishing communities. While reported whale entanglements remain higher than pre-2014 totals, reported whale entanglements in California have declined markedly in the years following the 2014–2016 large marine heatwave (Fig. 1b). This is a success for this fishery and attributed to increased awareness, development of best practices for fishing gear and the mitigation program to protect whales. We now need to be successful at protecting and mitigating the socio-economic impacts to fishery participants and the fishing communities they support.

## Methods

### The fishery

The California Dungeness crab fishery is divided into two management areas representing north and central (NMA and CMA, respectively), that have different opening dates. The Commercial Dungeness Crab fishing season typically spans from 1st December until 15th July in the NMA and from 15th November to 30th June in the CMA. The fishing season is named by calendar year in which the season ends (e.g. the 2020 season begins in late 2019 and continues into 2020). Open dates are sometimes adjusted due to crab meat quality, occurrence of harmful algal blooms or due to whale entanglement risk. Season open and close dates for each management area were compiled from online news reports and information provided by CDFW, then cross-checked with landings information following Richerson et al., 2020^62^.

The California commercial Dungeness crab fishery has an estimated 501 active participants based on permits^63^, however the number of vessels that have actively landed Dungeness crab between 2011 and 2020 ranges from 385 to 470 vessels in one season. The fishery has seen a rise in revenues in the past decade relative to the 2000–2010 period, following a sharp rise relative to the 1970–2000 period. In recent years the Dungeness crab fishery in California has seen annual ex-vessel revenues in excess of $80 million and landings in excess of 12,000 tons^54^. The abundance of pre-season legal size males has also increased greatly in the CMA, with 2000–2016 estimates of abundance averaging nearly five times greater than 1970–2000 estimates^62^. Abundance of pre-season legal size males in the NMA has been relatively stable. The fishery takes on average 83% and 65% of legal sized male crabs each year in the NMA and CMA, with the majority being landed in the first six weeks of the season^62^.

The 2019 season began on time in the CMA on 15 November 2018. However, opening was delayed in the NMA because of crab meat quality (i.e., low weight), and landings began on 22 January 2019. Both management areas were closed to fishing on 15 April due to whale entanglement risk. The season was therefore shortened in the NMA by 53 days (almost 8 weeks) due to a crab meat quality delay and a further 91 days (13 weeks) at the end of the season due to whale entanglement risk. There were additional zone specific delays due to domoic acid presence which delayed fishing up to 55 days at the beginning of the season in the Northern portion of the NMA. In the CMA, the season was shorted by 76 days (11 weeks) due to whale entanglement risk at the end of the 2019 season. The northern portion of the CMA also lost an additional 23 days at the beginning of the 2019 season due to domoic acid presence.

In the 2020 season, the NMA was again delayed due to crab meat quality until 31 December (30 days) but was not closed due to whale entanglement risk in the 2020 season. The CMA experienced both a delay at the beginning of the season until 15th December (30 days) due to persistence of humpback whales and Pacific leatherback turtles on fishing grounds and closed early on 15 May due to entanglement risk, 46 days before the official close date. The season was shortened in the CMA in the 2020 season by 76 days in total.

### Fishing and economic data

Data from individual vessel fish tickets (landings records) were provided by the California Department of Fish and Wildlife (CDFW) for all fisheries catches within the years 1981–2020. To include only fishing trips targeting this species, fish tickets recording catches of Dungeness crab were included in the analysis if gear specified “crab or lobster trap” or “entrapping”. Fifty-one duplicate fish tickets were removed and 357, 599 fish tickets reporting catches of Dungeness crab were included. Fish tickets specify the CDFW fishing block area (catch area code) where fishing took place and the port of landing. Fish tickets were assigned to the Northern or Central Management Area by CDFW fishing block (Fig. 2a). If the fishing block recorded spanned both management areas, or if fishing block information was missing, the latitude of the port of landing was used (Fig. 2a). The two management areas are divided at the Sonoma/Mendocino County line (the Central Management Area is < 38.77° latitude) (Fig. 2a-c).

All fish tickets recorded in one day by one vessel within one management area were counted as an individual fishing trip. Annual crab revenue per vessel was aggregated from fish tickets by vessel ID. Fish tickets with invalid vessel ID’s were removed prior to analysis. Only fish tickets with landing dates falling within open season dates for each management area were included when calculating within revenues within each respective management area.

### Model application

We follow the method introduced by Holland and Leonard (2020), used to predict California commercial Dungeness crab revenues during the 2016 season, which was disrupted by a long harmful algal bloom, and apply it to the same fishery during the 2019 and 2020 fishing seasons, which were interrupted by whale entanglement mitigation measures. Retrospective crab revenue estimates were calculated using a linear Cragg hurdle model (Stata15 TM)^38^. The hurdle model jointly estimates probability of participation, s_i_ (selection model) in the fishery and multiplies it by estimated revenue, h_i_, (revenues model) to predict the annual revenue per vessel^37^.

1 $${Y}_{i}= {s}_{i}{{h}_{i}}^{*}$$

where *s*_*i*_ is determined by the participation model:

2 $${s}_{i}= \left\{\begin{array}{c}1 if {z}_{i}Y+ {\epsilon }_{i}>0\\ 0\,otherwise\end{array}\right.$$

and the latent variable ${{h}_{i}}^{*}$ is observed if ${s}_{i}$=1:

3 $${{h}_{i}}^{*}={x}_{i}\beta +{v}_{i}$$

As per Holland and Leonard (2020)^38^, the selection model is a binary logit model of participation choice, while the revenue model is a linear model of annual revenue per vessel. The dependent variable for the selection model is participation, where the dependent variable is 1 if revenue > 0 and 0 otherwise. The dependent variable for the expected revenue model is observed annual vessel revenue from Dungeness crab. The model was fit with data from the 2011–2018 seasons (excluding 2016) to estimate revenues in the 2019 and 2020 seasons. This 7 year time period was chosen as a representative baseline period of levels of production in the recent history of the fishery given a change point in the 2011 season, especially in the CMA (Fig. 2c), in which catches by the fishery increased substantially and have since been sustained. We exclude the 2016 season from our analysis as a 5-month closure due to a harmful algal bloom severely reduced revenues in that season. We increase the baseline to 7 years, from the 5 years used by Holland and Leonard (2020), to increase the robustness of the model given this disturbance within our time period of interest. Vessels that had fished for Dungeness crab in California at least once during the 2011–2018 seasons were included.

Explanatory variables for the participation model (${z}_{i}$) and the conditional expected revenue model (${x}_{i}$) included mean revenue for the vessel the prior 7 years (Mean Crab Revenue), the mean percent of a vessels’ total revenue made up of Dungeness Crab in the prior 7 years (Mean Percent Crab), the mean latitude the vessels’ crab was landed in the prior 7 years weighted by revenue (Mean Latitude), the number of years the vessel fished in the prior 7 years (Years Fished) and an average index of diversification of a vessels diversification also over the prior 7 years (Concentration Index). The Herfindahl–Hirschman Index (HHI) was used as the index of the vessels’ diversification strategy, ranging from 0–1 where 0 is the highest diversity in catches (revenues) and 1 is the least diverse or concentrated (catches of just 1 species). We refer to this as a concentration index. These covariates averaged over the prior 7 years thus used “fish ticket” data from 2004 to 2010 to produce averages for the 2011 season, 2005–2011 to produce averages for the 2012 season and so on. Vessel length was included as a covariate as a proxy for characteristics such as vessel power and crew size which influence a vessels catch potential.

Following Holland and Leonard (2020)^31^, pre-season abundance estimates of Dungeness crab were included as an explanatory variable in both models. Retrospective abundance estimates using the method from Richerson et al.(2020)^62^, updated to the 2019/2020 season, were calculated using a hierarchical linear mixed-effects depletion estimator model. The model uses the trend in catch per unit effort (retained weight per trip) per vessel over the fishing season to estimate the biomass of legal sized male Dungeness crab at the beginning of the fishing season. Fish ticket data from 1981 onwards was used to estimate this model. An index of crab abundance separately for the NMA and CMA for each season was derived from the model (Fig. 2b,c) and included as an explanatory variable in the relevant hurdle model. Separate models were run for revenues in the Northern Management Area and Central Management Area of California due to differences in management in the two regions. An additional variable of “switching” was added to the ex-vessel revenues part of the hurdle model to account for vessels that fish in both management areas in a given season but do not have equal opportunity for fishing in both areas due to fair start rules which require a 30 day waiting period before transiting to land crab in another zone. We included this as a proxy variable of 0 = no switching behavior, 1 = vessel switched between two management areas in that season. Deviance explained by the model was calculated via an analogue to an R^2^ value as (1-(SSresidual/SStotal)) where SSresidual is the sum of squared difference between observed and predicted vessel revenues and SStotal is the sum of square differences between observed and mean vessel revenues^38^.

To estimate retrospective total fishery revenues that would have been observed in the absence of closures and other disturbances to the season, hurdle models predicted revenues for the 2019 and 2020 seasons for all vessels that participated in the fishery during the 2011–2018 seasons. Values predicted from the hurdle models were aggregated to the sum fishery total for the NMA and CMA. Estimated revenue losses were then calculated by subtracting the observed sum total of revenues for the 2019 and 2020 seasons from the predicted revenues. The 2019 and 2020 season’s revenues were predicted out-of-sample and therefore we provide a comparison of in-sample vs out-of-sample prediction of all years included within the model using a hold one out approach. To do this we iteratively held out one year of data, fit the model to the remaining data, then made predictions for the held-out year (Supplementary Table S2).

## Supplementary Information

Supplementary Information 1.

Supplementary Information 2.

## Data Availability

Data from individual vessel fish tickets (landings records) were provided by the California Department of Fish and Wildlife (CDFW). Vessel-level landings data are confidential and the raw data cannot be made public. Data is available by CDFW upon request to CDFW (Paulo Serpa, Paulo.Serpa@wildlife.ca.gov).
