# Supplementary material for: Revenue loss due to whale entanglement mitigation and fishery closures
Source: Sci Rep. 2022 Dec 13;12:21554. doi: 10.1038/s41598-022-24867-2 (PMC9746587; doi:10.1038/s41598-022-24867-2)
Supplement: Supplementary file 1 — Supplementary Information 1. [file 41598_2022_24867_MOESM1_ESM.docx]

# Revenue loss due to whale entanglement mitigation and fishery closures

Rachel Seary*^abc^, Jarrod A. Santora^bd^, Desiree Tommasi^ae^, Andrew Thompson^e^, Steven Bograd^ac^, Kate Richerson^f^, Stephanie Brodie^ab^, Dan Holland^g^

^a^Institute of Marine Sciences, University of California, Santa Cruz, California, USA

^b^Fisheries Ecology Division, Southwest Fisheries Science Center, National Marine Fisheries Service, National Oceanic and Atmospheric Administration, Santa Cruz, CA, USA

^c^Environmental Research Division, Southwest Fisheries Science Center, National Marine Fisheries Service, National Oceanic and Atmospheric Administration, Monterey, CA, USA

^d^Department of Applied Math, University of California, 1156 High Street, Santa Cruz, California 95064, USA

^e^Fisheries Research Division, Southwest Fisheries Science Center, National Marine Fisheries Service, National Oceanic and Atmospheric Administration, San Diego, CA, USA

^f^Fishery Resource Analysis and Monitoring Division, Northwest Fisheries Science Center Newport Field Station, 2032 S.E. OSU Drive, Newport, OR 97365, USA

^g^Conservation Biology Division, Northwest Fisheries Science Center, 2725 Montlake Blvd E., Seattle, WA 98112, USA

**Supplementary materials**

Supplementary Table 1. Coefficient estimates from a linear Cragg hurdle model of California commercial Dungeness crab fishery revenues in North (NMA) and Central California (CMA). The dependent variable was annual ex-vessel revenue from Dungeness Crab (thousand US dollars) and covariates included were the mean annual crab revenue (ex-vessel revenue in thousand US dollars) that a vessel reported over the baseline period, the mean percent of Dungeness Crab in a vessels total annual catch, the number of years a vessel has fished during the baseline period, an estimate of pre-season crab abundance (N.CA or C.CA Crab Index), the mean latitude of catches, vessel length and the Herfindahl-Hirschman Index (HHI) of catch diversity. The HHI ranges from 0-1 where 0 is the highest diversity in catches and 1 is the least diverse (catches of just 1 species). We also used a binary variable (0 or 1) for vessels that switch between both management areas within a given season for the revenues model only. The selection model represents probability of participation in the fishery, while the crab revenue model represents revenue conditional on participation.

| Variable | Selection Model Coefficient |  | P-Value |  | Crab Revenue Model Coefficient |  | P- Value |  |
| --- | --- | --- | --- | --- | --- | --- | --- | --- |
|  | **NMA** | **CMA** | **NMA** | **CMA** | **NMA** | **CMA** | **NMA** | **CMA** |
| Mean Crab Revenue | 0.003 | 0.002 | 0.000 | 0.000 | 1.135 | 1.215 | 0.000 | 0.000 |
| Mean Percent Crab | -0.004 | 0.007 | 0.167 | 0.000 | 1.688 | 1.800 | 0.105 | 0.004 |
| Years Fished | 0.291 | 0.226 | 0.000 | 0.000 | 23.122 | 9.163 | 0.000 | 0.002 |
| Diversification (HHI) | -0.645 | -0.559 | 0.044 | 0.019 | -204.438 | -147.551 | 0.052 | 0.033 |
| N.CA or C.CA Crab Index | 0.067 | 0.119 | 0.000 | 0.000 | 61.553 | 35.578 | 0.000 | 0.000 |
| Mean Crab Latitude | -0.140 | -0.028 | 0.000 | 0.502 | 34.006 | -30.093 | 0.003 | 0.010 |
| Vessel Length | -0.012 | -0.010 | 0.000 | 0.000 | 5.233 | 9.982 | 0.000 | 0.000 |
| Switching |  |  |  |  | -61.891 | -22.790 | 0.005 | 0.169 |
| Constant | 5.497 | 0.000 | 0.000 | 1.000 | -2232.76 | 281.267 | 0.000 | 0.516 |

Supplementary Figure 1. Model residual plots showing (a-b) Predicted vs observed vessel level Dungeness crab revenue, (c-d) Residual crab revenue (observed-predicted) vs observed crab revenues and (e-f) boxplots of prediction residuals across seasons for the Northern Management Area (NMA) and Central Management Area (CMA) of California.


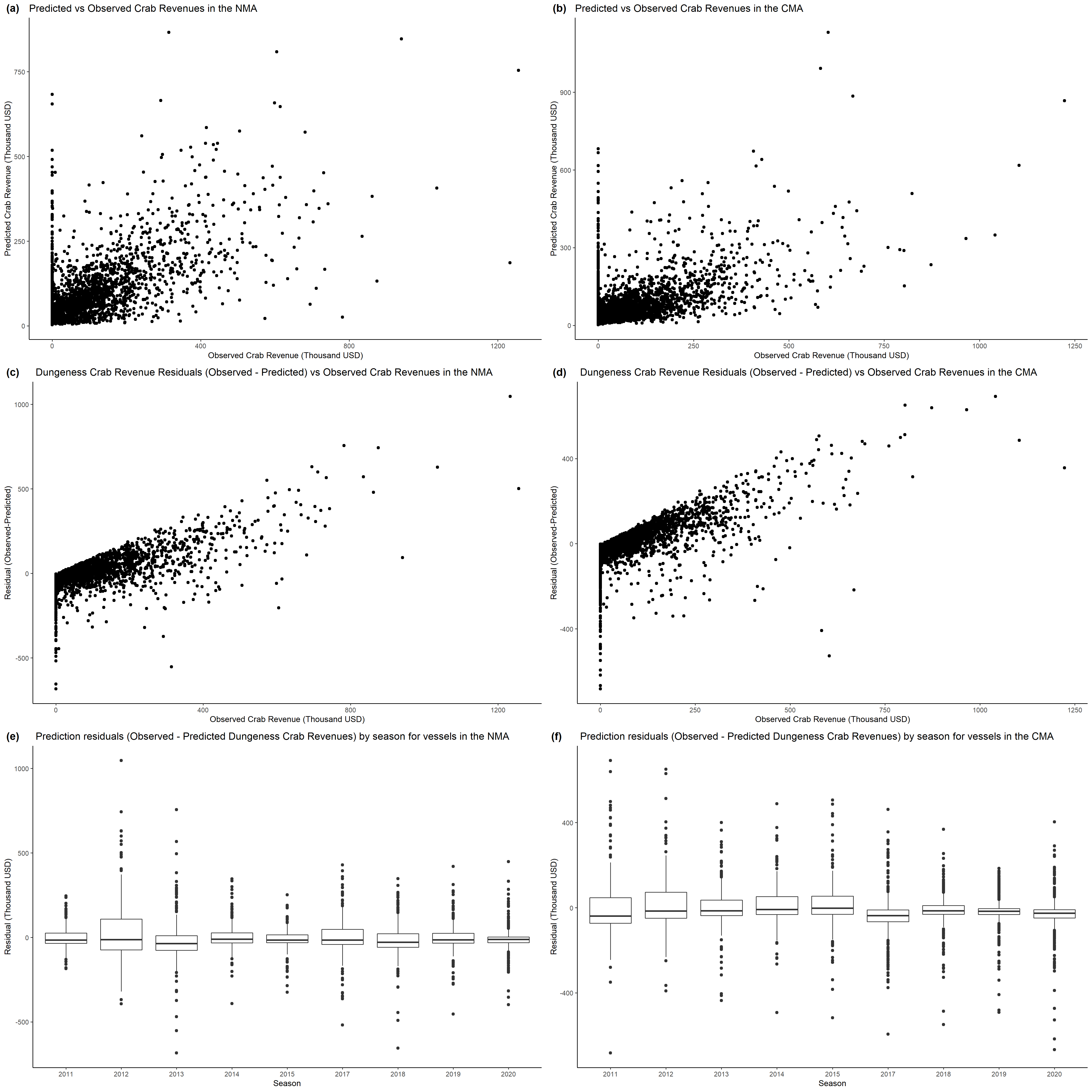


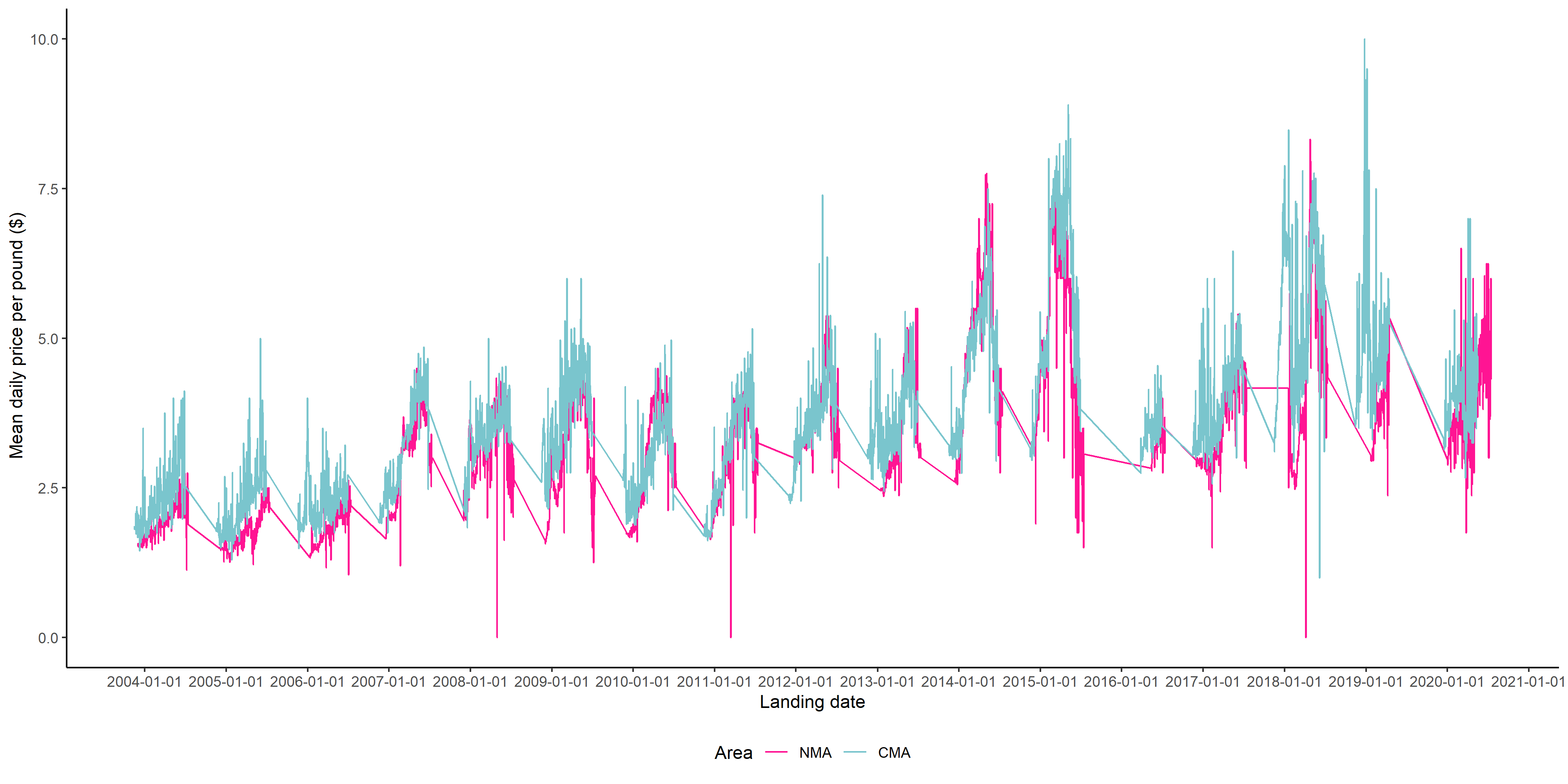
Supplementary Figure 2. Mean price per day of Dungeness crab landed at California fishing ports in the northern and central management areas from the 2011 to 2020 fishing seasons. Note that during 2020 when the CMA closed early due to whale entanglement risk, the NMA continued to show increasing price per pound, possibly indicating continued demand for crab during the COVID-19 pandemic.

Supplementary Table 2. Comparison of in-sample to out of sample prediction of Dungeness crab revenues (million US dollars) for the Northern and Central Management Areas of the commercial Dungeness crab fishery in California. Out of sample predictions were calculated using a leave one out approach (e.g. data for the 2011 season was removed from the model to predict 2011 revenues). Model residuals are calculated as observed revenue - predicted revenue. An analogue to an R^2^ was calculated for each of these predictions by season as described in the methods section of the manuscript.

| **Season** | **Observed Revenue** | **In sample prediction (original model)** | **Residual**  **(in sample)** | **Analogue R^2^**  **(in sample)** | **Out of sample prediction** | **Residual**  **(out of sample)** | **Analogue R^2^**  **(out of sample)** |
| --- | --- | --- | --- | --- | --- | --- | --- |
| **Northern Management Area** | | | | | | | |
| 2011 | 18.257 | 18.258 | -0.001 | 0.527603672 | 18.285 | -0.028 | 0.468392 |
| 2012 | 48.799 | 39.024 | 9.775 | 0.32195103 | 35.156 | 13.643 | 0.257956 |
| 2013 | 40.913 | 46.559 | -5.646 | 0.344875896 | 49.512 | -8.599 | 0.335459 |
| 2014 | 22.296 | 20.777 | 1.519 | 0.304557056 | 19.991 | 2.305 | 0.30627 |
| 2015 | 12.812 | 16.827 | -4.015 | -0.141524937 | 19.102 | -6.29 | -0.3695 |
| 2017 | 32.99 | 30.721 | 2.269 | 0.254133367 | 29.89 | 3.1 | 0.193523 |
| 2018 | 42.739 | 49.951 | -7.212 | 0.443276318 | 53.079 | -10.34 | 0.378439 |
| **Average** |  |  | **-0.473** | **0.2935532** |  | \| **-0.887** \| \| --- \| | **0.224362** |
| **Central Management Area** | | | | | | | |
| 2011 | 35.006 | 34.497 | 0.509 | 0.285734735 | 33.867 | 1.139 | 0.261827671 |
| 2012 | 34.955 | 29.411 | 5.544 | 0.378684578 | 28.422 | 6.533 | 0.34706945 |
| 2013 | 22.612 | 21.986 | 0.626 | 0.085097426 | 21.673 | 0.939 | 0.052207437 |
| 2014 | 33.156 | 28.008 | 5.148 | 0.324466412 | 27.094 | 6.062 | 0.307030511 |
| 2015 | 40.871 | 34.181 | 6.69 | 0.351354678 | 32.66 | 8.211 | 0.338447007 |
| 2017 | 33.072 | 49.868 | -16.796 | 0.308299039 | 55.291 | -22.219 | 0.216024515 |
| 2018 | 21.062 | 27.077 | -6.015 | 0.079432936 | 32.741 | -11.679 | -0.189711918 |
| **Average** |  |  | **-0.6134** | **0.259009972** |  | **-1.57342** | **0.190413525** |

Supplementary Table 3. Five year average revenues (million US Dollars) in the California commercial Dungeness crab fishery as they would be used under typical disaster assistance calculations to predict 2019 and 2020 season revenues.

| Season | Historical seasons | 5 year average revenue | Observed revenue | Residual |
| --- | --- | --- | --- | --- |
| 2019 | 2014-2018 | 59.99 | 47.3 | -10.62 |
| 2020 | 2015-2019 | 57.39 | 46.8 | -12.73 |
